# Serum and Clinical Factors Associated with Total Knee Arthroplasty in Patients with Knee Osteoarthritis

**DOI:** 10.3390/life16020232

**Published:** 2026-02-01

**Authors:** Sergiu Andrei Iordache, Adrian Cursaru, Bogdan Serban, Mihnea Ioan-Gabriel Popa, Mihai Aurel Costache, Sergiu Stanciu, Bogdan Stefan Cretu, Florin Catalin Cirstoiu

**Affiliations:** 1 Department of Orthopedics, Carol Davila University of Medicine and Pharmacy, 050474 Bucharest, Romania; sergiu-andrei.iordache@drd.umfcd.ro (S.A.I.); serbann.bogdan@yahoo.com (B.S.); mihnea.ioan.popa90@gmail.com (M.I.-G.P.); mihaiaurelcostache@yahoo.com (M.A.C.); jfrbogdan@yahoo.com (B.S.C.); cirstoiu_catalin@yahoo.com (F.C.C.); 2 Orthopedic Department, Bucharest University Emergency Hospital, 050098 Bucharest, Romania; stanciu.sergiu98@gmail.com

**Keywords:** knee osteoarthritis (KOA), total knee arthroplasty (TKA), serum biomarkers, Interleukin-6 (IL-6), cartilage oligomeric matrix protein (COMP)

## Abstract

Knee osteoarthritis (KOA) is one of the most prevalent chronic joint disorders, with its incidence rising over the past decade due to an increase in risk factors, including age, obesity, metabolic conditions, sedentary behavior, and mechanical stress on the knee joints. We conducted a cross-sectional, two-group comparison including 70 knee-pain patients aged ≥ 44 years: 50 patients meeting clinical and radiological criteria for TKA and 20 patients undergoing knee arthroscopy as controls. All patients underwent clinical assessments, WOMAC scoring, radiography, and 3T knee MRI. Serum interleukin-6 (IL-6), cartilage oligomeric matrix protein (COMP), vitamin D3, calcium, and phosphorus were measured at admission. TKA patients were older and had higher WOMAC scores. WOMAC discriminated groups perfectly (AUC = 1.000), but age discriminated well (AUC = 0.911). IL-6 (AUC = 0.819) and COMP (AUC = 0.838) were significant discriminators, with IL-6 threshold ≥ 4.585 pg/mL achieving 66% sensitivity and 85% specificity and COMP cutoff ≥ 11.52 ng/mL achieving 84% sensitivity and 75% specificity. TKA group vitamin D3 levels were considerably lower but had limited discriminatory performance (AUC = 0.683). Although all patients had adequate mineral metabolism, TKA patients had lower serum calcium and higher serum phosphorus levels than controls.

## 1. Introduction

Knee osteoarthritis (KOA) is one of the most prevalent chronic joint disorders, with its incidence rising over the past decade due to an increase in risk factors, including obesity, metabolic conditions, sedentary behavior, and mechanical stress on the knee joints, along with various modifiable and non-modifiable factors that contribute to articular cartilage degeneration through pathophysiological mechanisms that remain incompletely elucidated [1,2,3].

Given the high prevalence of knee osteoarthritis, early diagnosis is crucial, relying on both clinical and paraclinical examinations [4]. From a clinical point of view, patients with KOA report pain exacerbated by knee joint overload, stiffness and swelling of the knee, a sensation of knee locking, but also the gradual loss of normal mobility of the knee joint, all of which may be the cause of quadriceps muscle hypotrophy [5]. The WOMAC score (The Western Ontario and McMaster Universities Arthritis) is an index utilized globally to evaluate the progression of knee osteoarthritis, comprising 24 elements associated with pain, knee joint stiffness, and physical function [6]. And from a paraclinical perspective, laboratory analyses such as COMP (Cartilage Oligomeric Matrix Protein), Il-6 (Interleukin 6), MMP (matrix metalloproteinases), CRP (C-reactive protein), Vitamin D3, serum calcium and phosphorus, parathyroid hormone levels, can be used. Additionally, imaging examinations, the most effective method for measuring the extent of knee osteoarthritis (KOA) is radiography in two views: anteroposterior and lateral [7].

Knee osteoarthritis can be considered an inflammatory pathology, characterized by an imbalance of the articular homeostatic environment due to various risk factors, leading to a progressive process where the articular cartilage loses its ability to regenerate to the initial state or adapt to the current condition [8,9,10]. Thus, this imbalance can be evaluated by measuring the level of certain specific biomarkers in the blood, urine or synovial fluid, providing essential information about the evolution of knee osteoarthritis [10,11]. This can be explained by the activation of caspases, through the process of cell apoptosis, leading to the activation of the inflammatory process, resulting in high levels of inflammatory cytokines in patients with knee osteoarthritis, such as Il-1B, Il-6, TNF-alpha [10,12]. Certain biomarkers determined in the synovial fluid are COMP and CTX-II, with the gradual degradation of the articular cartilage demonstrating a strong association between the increased level of COMP and CTX-II and the severity of TKA [13,14,15]. Certainly, a single biomarker is not very specific regarding the diagnosis and evolution of knee osteoarthritis; however, the association of several biomarkers helps to diagnose and monitor the progression of knee osteoarthritis [10,16].

The treatment of knee osteoarthritis could be either conservative or surgical. Most patients present to the orthopedic doctor when the pain prevents them from carrying out their daily lives, so the administration of analgesic and anti-inflammatory treatment is recommended in the initial phase [17]. Oral NSAIDs (e.g., naproxen, ibuprofen, celecoxib) represent the first step of treatment, but their therapeutic benefit must be balanced against their risk of cardiovascular, gastrointestinal or renal adverse reactions; therefore, it is recommended to administer them for a limited period of time and at the lowest effective dosage [18,19]. In cases where patients are unable to take NSAIDs, specialist research has demonstrated that Tramadol or alternative opioids are effective for pain management; nevertheless, these medications also have certain hazards, particularly with prolonged usage [20]. However, despite the use of these anti-inflammatory and analgesic treatments, it has been demonstrated that they address only the symptoms of the disease rather than its underlying cause, delaying the time of surgery [21].

Metabolically, knee osteoarthritis may be associated with parathyroid hormone levels, which are involved in activating osteoclasts, promoting bone resorption and releasing calcium and phosphorus, maintaining their homeostasis [22]. Numerous studies have demonstrated the anabolic effect of PTH on bone, which has 2 beneficial functions in knee osteoarthritis, namely anti-inflammatory and anti-senescence [22,23]. The impact of PTH levels in TKA is it considered to be influenced by serum calcium levels, with specialized research showing an association between elevated calcium levels and an increase in the progression of knee osteoarthritis [23]. Although these results are promising, further study is necessary to draw a conclusion [24]. Vitamin D may influence the progression of knee osteoarthritis through the expression of VDR (vitamin D receptor) in articular cartilage in patients with KOA, as well as through the endocrine system mentioned above [25,26]. These could all be alternative methods for diagnosing and measuring the condition, but further research is required to fully understand their role in the evolution of knee osteoarthritis.

Despite the growing emphasis on early diagnosis and prognostic classification in knee osteoarthritis research, numerous potential biomarkers have been assessed primarily in cross-sectional clinical contexts rather than in longitudinal cohorts. In these instances, correlations with illness severity or surgical decision-making must be differentiated from accurate predictions of future disease progression. Consequently, biomarkers assessed at presentation may indicate the present inflammatory and degenerative load rather than long-term risk.

The objective of this study was not to evaluate the longitudinal progression to total knee arthroplasty, but to investigate the association of admission-time clinical scores, imaging results, and specific serum biomarkers with the classification of patients as appropriate for total knee arthroplasty versus those suitable for arthroscopy in a symptomatic knee osteoarthritis cohort.

Patients receiving knee arthroscopy were chosen as a comparator group due to their representation of a clinically pertinent population experiencing knee discomfort and structural disease necessitating surgical intervention, albeit at a less advanced level than those indicated for arthroplasty. Although arthroscopy is not a neutral control in knee osteoarthritis, its use facilitated a comparison between two divergent treatment modalities based on symptom severity, functional impairment, and imaging results upon presentation. The authors recognize that age and symptom load vary among groups and are inherently connected to surgical assignment.

## 2. Materials and Methods

The study was conducted from 1 January 2023 to 31 March 2024. The inclusion criteria consisted of individuals aged over 44 years experiencing knee discomfort who presented to the hospital’s orthopedic department. The standard diagnostic protocol included a clinical examination, computation of the WOMAC score, standardized weight-bearing anteroposterior and lateral knee radiographs for all patients. Radiographic severity was assessed qualitatively by experienced orthopedic surgeons; formal Kellgren–Lawrence grading was applied and 3 Tesla knee MRI was performed. The patient group included 70 patients and was divided into two groups: one consisting of patients meeting clinical and radiological criteria for total knee arthroplasty (n = 50) and a control group requiring knee arthroscopy (n = 20). Exclusion criteria were acute joint trauma, signs of osteo-articular infection, patients known to have autoimmune diseases and joint damage, inability to perform 3 Tesla MRI, presence of other diseases that could modify the measured parameters, inability to obtain all clinical data or loss to follow-up. For each patient, the serum value of Interleukin 6 (N value ≤ 7 pg/mL), vitamin D3 (N value = 30–100 ng/mL), Serum Calcium (N value = 8.5–10.5 mg/dL), Serum Phosphorus (N value = 2.5–4.5 mg/dL) and Cartilage Oligomeric Matrix Protein (COMP) (N value ≤ 12 ng/mL) were measured upon admission. Indication for total knee arthroplasty was based on a combination of severe clinical symptoms refractory to conservative treatment, radiographic evidence of advanced osteoarthritis, functional limitation affecting daily activities, and reduced knee range of motion. Conservative treatment failure was defined as persistent symptoms despite at least six months of non-operative management. WOMAC scores and analgesic consumption were recorded at admission for research purposes and were not used as formal inclusion criteria or predefined thresholds for surgical allocation, although symptom severity inherently contributes to clinical decision-making.

Analgesic usage was measured by the number of days on which at least one dose was required for the effective execution of personal activities. The primary outcome was indication status for total knee arthroplasty at presentation, defined by multidisciplinary orthopedic assessment, and not longitudinal progression to surgery. Statistical analysis was performed using IBM SPSS Statistics 27, MedCalc Software Ltd., Ostend, Belgium (Version 23.2.1), Excel 2019 MSO (Version 2503) and GraphPad Prism version 10.4.1. The statistical methods and tests used in the analysis were represented by Multivariate Linear Regression, Binary Logistic Regression, Independent Samples *t*-test (t), Mann—Whitney U test (U), One-way Analysis of Variance with Repeated Measures (ANOVA) (F), Friedman test (χ^2^), Chi—square test (χ^2^), Fisher test, Phi correlation coefficient (φ), Cramer’s V correlation coefficient, Pearson coefficient (r), Spearman correlation index (rs), Youden coefficient (J). Normality of distributions was established using Kolmogorov–Smirnov and Shapiro–Wilk tests, as well as graphical analysis. Levene’s test was used to check the homogeneity of distributions, Durbin Watson test was used to check the independence of errors, and the Mauchly test was used to check sphericity (with a Greenhouse-Geisser correction). Homoscedasticity and linearity were determined by graphical analysis. A multivariable logistic regression model including a limited number of clinically relevant variables was considered the primary analytical approach, while univariable analyses and ROC curve assessments were exploratory and hypothesis-generating. Given the sample size, no formal correction for multiple testing was applied

Study approval was granted from the local ethics committee of the University Emergency Hospital of Bucharest, and informed consent was obtained from all participants.

## 3. Results

Patients undergoing total knee arthroplasty utilized analgesics for a duration ranging from 86 to 133 days in the six months prior to the treatment, with a mean of 108.86 days (standard deviation: 11.85; CI: [105.49; 112.22]). The median duration of consumption was 109.5 days.

Patients undergoing arthroscopy utilized analgesics for a duration ranging from 40 to 75 days in the six months prior to the operation, with a mean of 50.15 days (standard deviation: 10.50; CI: [45.23; 55.06]). The median duration of consumption was 46.5 days.

**Figure 1:**
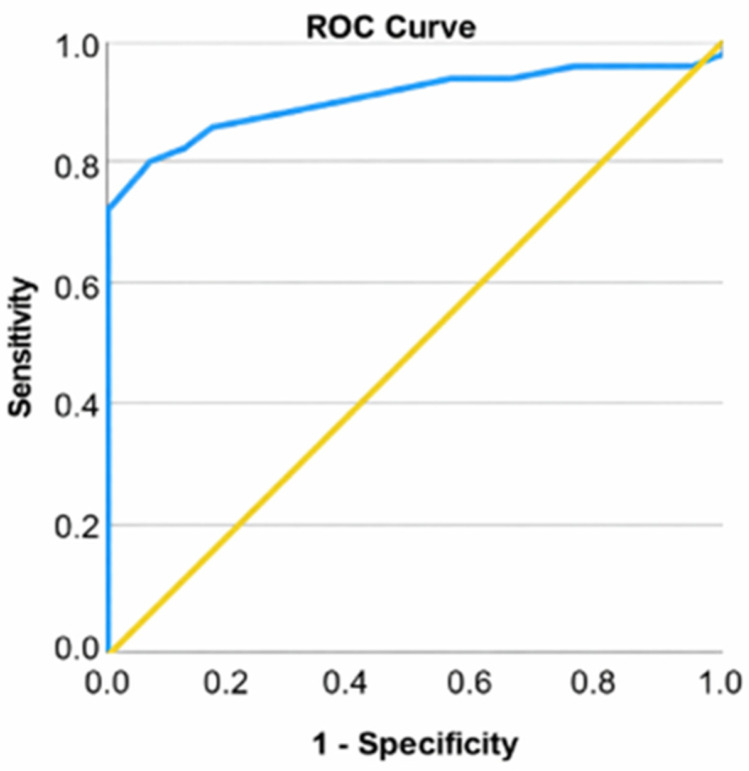
ROC curve for age and arthroplasty.

Patients experiencing total knee arthroplasty were markedly older (65.12 ± 8.19 years) compared to those requiring arthroscopy (52.55 ± 3.63 years), t = −8.882, *p* ≤ 0.001. The calculation of the odds ratio (OR) revealed that advanced age conferred a 1.305-fold or 30.5% increased likelihood of patients with KOA requiring total knee arthroplasty (OR = 1.305, CI: [1.141; 1.493]). ROC curve to assess the effectiveness of age in categorizing patients based on the kind of surgery (total knee arthroplasty or arthroscopy). Age was confirmed as a significant predictor of the necessity for arthroplasty (*p* ≤ 0.001), with an area under the curve (AUC) value of 0.911 demonstrating exceptional discrimination among intervention modalities (Figure 1).

Among patients with KOA, women constitute 67.14% of the examined cases (n = 47, with 2 out of 3 patients being female), while men account for 32.86% (n = 23). A balanced distribution between the sexes was noted in the arthroscopy group, with 55% conducted on men (n = 11) and 45% on women (n = 9).

**Figure 2:**
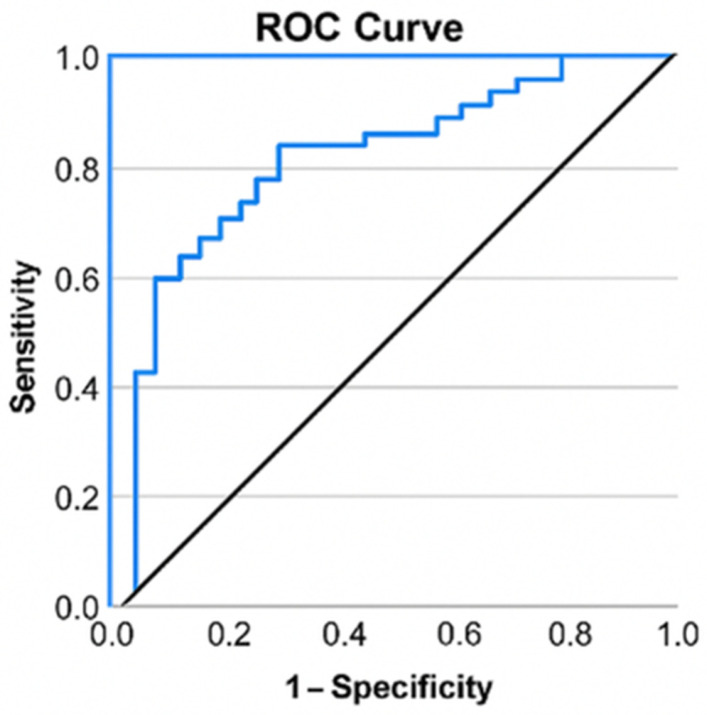
ROC curve for WOMAC score and total knee arthroplasty.

Patients undergoing total knee arthroplasty exhibited WOMAC scores between 47 and 89, with a mean of 66.28 (standard deviation: 10.81; confidence interval: [63.07; 69.49]) and a median of 65.00. Patients undergoing arthroscopy had WOMAC scores between 13 and 31, with a mean of 20.25 (standard deviation: 5.36; CI: [17.77; 22.73]) and a median of 20.00. A *t*-test was conducted to ascertain variations in WOMAC scores between individuals undergoing arthroscopy and those receiving total knee arthroplasty. Patients requiring total knee arthroplasty exhibited a higher WOMAC score (66.28 ± 10.81) compared to those undergoing arthroscopy (20.25 ± 5.36), with a mean difference of 46.03 points that was statistically significant, t = −23.696, *p* < 0.001. The ROC curve was used to evaluate the efficacy of the initial WOMAC score in categorizing patients based on the type of surgery (total knee arthroplasties or arthroscopies) (Figure 2).

**Figure 3:**
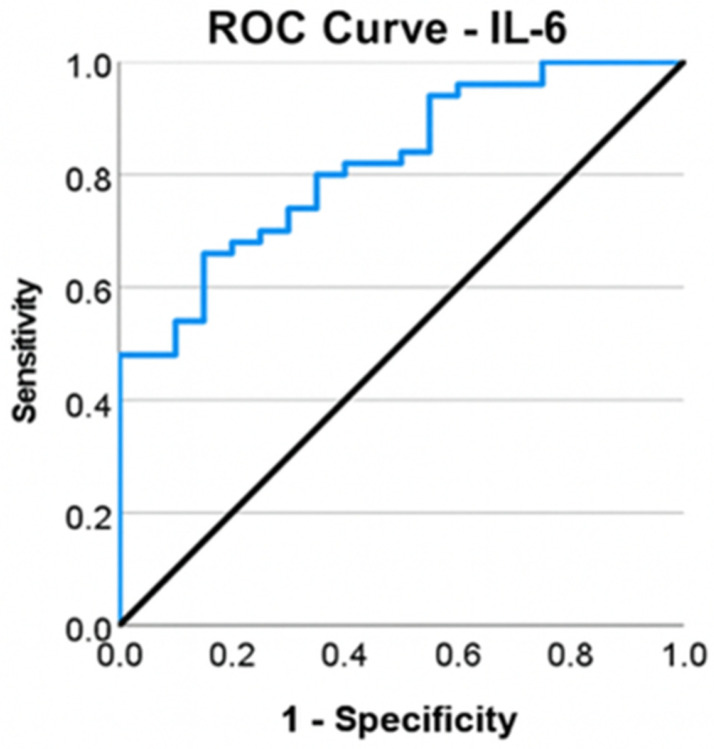
ROC curve for IL-6 and total knee arthroplasty.

A ROC curve was employed to evaluate the efficacy of IL-6 in categorizing patients based on the type of surgery (total knee arthroplasty or arthroscopies). Interleukin-6 (IL-6) showed moderate-to-good discrimination between the TKA and arthroscopy groups when analyzed individually (*p* ≤ 0.001), exhibiting an area under the curve (AUC) value of 0.819, which demonstrates a robust capacity to differentiate between intervention types (Figure 3).

The Youden index was computed to determine the appropriate cutoff for IL-6. The coefficient’s maximum value (J = 0.51) was achieved at an IL-6 threshold of ≥4.585 pg/mL, yielding a sensitivity of 66% and a specificity of 85%.

Patients undergoing total knee arthroplasty exhibited COMP values ranging from 9.52 to 19.03 ng/mL, with a mean of 13.24 ng/mL (standard deviation: 2.02; 95% confidence interval: [12.69; 13.79]) and a median of 13.09 ng/mL.

**Figure 4:**
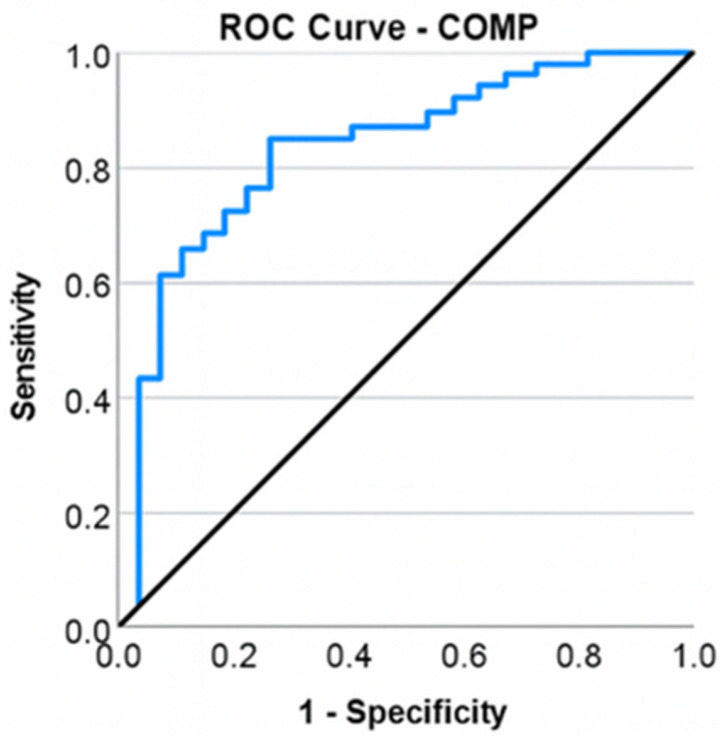
ROC curve for COMP and total knee arthroplasty.

Arthroscopy patients exhibited COMP values ranging from 7.79 to 13.86 ng/mL, with a mean of 10.85 ng/mL (standard deviation: 1.57; confidence interval: [10.19; 11.51]) and a median of 11.26 ng/mL. Applying a Chi-square test, it was observed that patients with elevated COMP levels were moderately more likely to undergo total knee arthroplasty compared to those with normal levels. Patients requiring total knee arthroplasty exhibited significantly higher frequencies of elevated COMP levels, χ^2^ = 13.263, φ = 0.435, *p* < 0.001. An elevated COMP level significantly increases the likelihood of the patient requiring a total knee arthroplasty by a factor of 8.5, OR = 8.5, CI: [2.44; 29.56], *p* ≤ 0.001. COMP showed moderate-to-good discrimination between the TKA and arthroscopy groups when analyzed individually (*p* ≤ 0.001), with an area under the curve (AUC) of 0.838, reflecting a highly effective capacity to differentiate between types of interventions (Figure 4). The Youden coefficient was determined to ascertain the optimal COMP cutoff. The highest coefficient value (J = 0.590) was achieved at a COMP cutoff of ≥11.52 ng/mL, corresponding to a sensitivity of 84% and a specificity of 75%.

Patients undergoing total knee arthroplasty exhibited vitamin D levels between 11.23 and 37.16 ng/mL, with a mean of 19.26 ng/mL (standard deviation: 5.24; confidence interval: [18.27; 20.25]) and a median of 18.83 ng/mL. Merely 4% of individuals exhibited optimum vitamin D levels (n = 2, 1 out of 25 patients).

Patients undergoing arthroscopy exhibited vitamin D levels between 12.87 and 34.32 ng/mL, with a mean of 22.20 ng/mL (standard deviation: 5.46; confidence interval: [20.34; 24.06]) and a median of 23.58 ng/mL. Merely 5% of individuals exhibited optimum vitamin D levels (n = 1, 1 out of 20 patients).

The Mann–Whitney U test revealed that patients undergoing total knee arthroplasty exhibited significantly lower vitamin D levels compared to those undergoing arthroscopy, with deficiency present in both groups, U = 317.500, Z = −2.373, *p* = 0.018.

**Figure 5:**
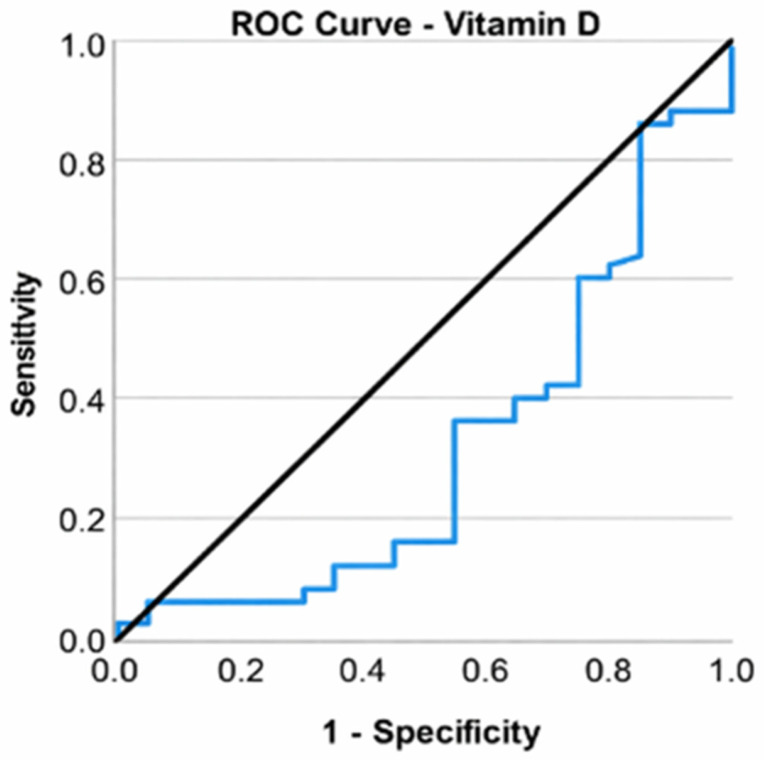
ROC curve for vitamin D and total knee arthroplasty.

Elevating vitamin D levels resulted in a 9.6% reduction in the likelihood of patients with KOA necessitating total knee arthroplasty (OR = 0.904, CI: [0.818; 1], *p* = 0.049). A receiver operating characteristic (ROC) curve was used to assess the ability of vitamin D to classify patients according to the type of surgery (total knee arthroplasty or arthroscopic surgery). Vitamin D was found to be a valid predictor of the need for arthroplasty (*p* = 0.018), but the area under the curve (AUC) value of 0.683 indicated a poor ability to discriminate between types of interventions. An optimal cutoff could not be chosen in this case (Figure 5).

Patients undergoing total knee arthroplasty exhibited blood calcium levels between 8.50 and 9.41 mg/dL, with a mean of 8.80 mg/dL (standard deviation: 0.29; confidence interval: [8.73; 8.87]) and a median of 8.76 mg/dL. All patients had normal levels of the measure.

**Figure 6:**
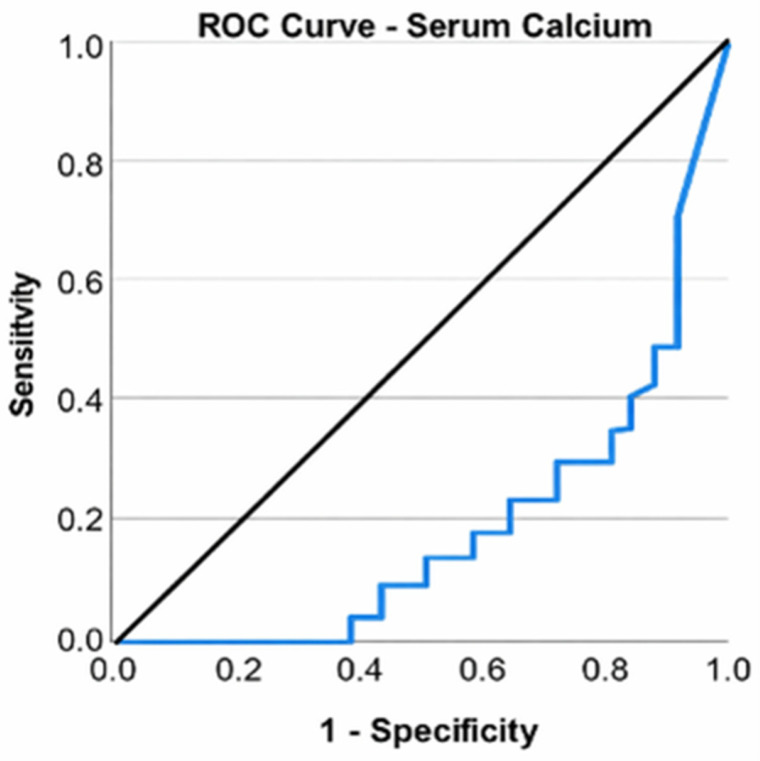
ROC curve for serum calcium level and total knee arthroplasty.

Patients undergoing arthroscopy exhibited blood calcium levels between 8.50 and 10.29 mg/dL, with a mean of 9.29 mg/dL (standard deviation: 0.49; CI: [9.04; 9.54]) and a median of 9.20 mg/dL. All patients had normal levels of the measure. Despite all patients exhibiting normal serum calcium levels, a Mann–Whitney U test revealed that those undergoing total knee arthroplasty had significantly lower values than those undergoing arthroscopy, U = 202.500, Z = −3.896, *p* ≤ 0.001. A ROC curve was used to assess the ability of serum calcium to classify patients according to the type of surgery (total knee arthroplasty or arthroscopic surgery). Serum calcium was found to be a valid predictor of the need for arthroplasty (*p* ≤ 0.001); the area under the curve (AUC) value of 0.798 indicated a good ability to discriminate between types of interventions. An optimal cutoff could not be calculated (Figure 6).

Patients undergoing total knee arthroplasty exhibited serum phosphorus levels between 3.36 and 4.50 mg/dL, with a mean of 3.95 mg/dL (standard deviation: 0.29; CI: [3.94; 3.97]) and a median of 3.94 mg/dL. All individuals exhibited normal serum phosphorus concentrations.

**Figure 7:**
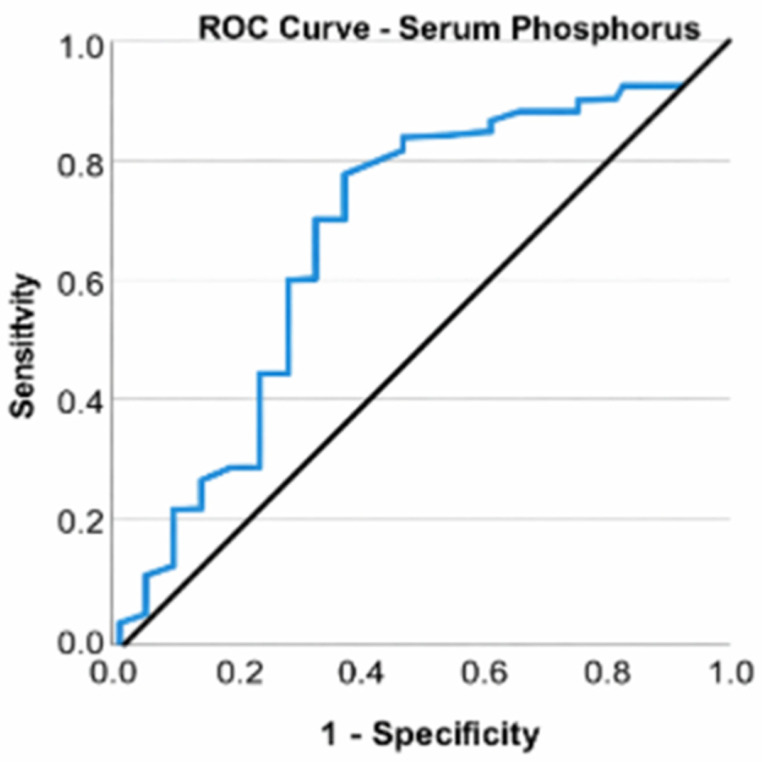
ROC curve for serum phosphorus and total knee arthroplasties.

Patients undergoing arthroscopy exhibited blood phosphorus levels between 2.82 and 4.37 mg/dL, with a mean of 3.67 mg/dL (standard deviation: 0.37; CI: [3.60; 3.74]) and a median of 3.60 mg/dL. All individuals exhibited normal serum phosphorus concentrations. Despite all patients exhibiting normal serum phosphorus levels, a Mann–Whitney U test revealed that those undergoing total knee arthroplasty had significantly elevated values compared to those undergoing arthroscopy, U = 738.500, Z = −3.095, *p* = 0.002. Elevated phosphorus levels, even within normal ranges, result in over a 17-fold increase in the likelihood of patients with KOA necessitating total knee arthroplasty (OR = 17.569, CI: [2.438; 126.611], *p* = 0.004). A ROC curve was used to assess the ability of serum phosphorus to classify patients according to the type of surgery (total knee arthroplasty or arthroscopies). Serum phosphorus was found to be a valid predictor of the need for arthroplasty (*p* = 0.002), with an area under the curve (AUC) value of 0.738 indicating a good ability to discriminate between types of interventions (Figure 7).

We have listed the primary biomarkers examined along with their statistical outcomes (Table 1).

## 4. Discussion

Identifying serum markers in patients with KOA is crucial due to the increasing frequency of this disorder and its medical, social, and economic impacts on national health systems. Total knee arthroplasty, the only surgical intervention aimed at treating patients with knee osteoarthritis, promotes pain relief and mobility restoration, yet is associated with risks. TKA represents the final stage in the evolution of a patient with KOA; thus, all variables assessed in this study pertain to the risk of necessitating total knee arthroplasty, reflecting the ineffectiveness of conservative treatments and the advancement towards the terminal phase of the disease. The preventive medical approach and the prevalence of screening programs have increased interest in the utility of prognostic indicators and the identification of at-risk patients within the medical community.

The WOMAC score, combined with age, has been shown as a strong predictor of the necessity for total knee arthroplasty, as evidenced by the research conducted by Bianca Prevot L et al., which analyzed 7552 knees from the multicenter Osteoarthritis Initiative (OI) database [27].

Interleukin-6, an inflammatory biomarker, has been identified as a predictor for the necessity of total knee arthroplasty, with a threshold value of >4585 pg/mL. The sensitivity and specificity of this marker indicate that it may also be elevated in other inflammatory or infectious disorders; thus, without appropriate screening for alternative pathologies, its value may provide false positives. Livshits G et al. [28] discovered IL-6 as a prognostic indicator of imaging progression in knee osteoarthritis (KOA), hence necessitating complete knee arthroplasty. The finding of COMP as a predictor of TKA in patients with KOA is attributed to increased articular cartilage degradation, as demonstrated by Lambova SN et al. [29] in a study including obese patients with KOA, linking elevated levels in obese individuals to increased mechanical joint stress. The proposed IL-6 and COMP cutoff values require validation in larger, prospective longitudinal studies before being considered for clinical application. Vitamin D3, in conjunction with modified mineral metabolism in individuals with knee osteoarthritis (KOA), as indicated by serum calcium and phosphorus levels, is directly associated with clinical or radiographic progression of KOA. Zhang FF et al. demonstrated that vitamin D deficiency is linked to the progression of KOA in a cohort of 418 participants. However, there are currently no prospective studies involving substantial patient groups to validate the predictive capacity of serum phosphorus and calcium in the disease’s evolution [30]. Future studies are warranted to develop and validate integrated multivariable models incorporating clinical, imaging, and biochemical parameters.

## 5. Conclusions

In this cross-sectional cohort of symptomatic knee osteoarthritis patients, age and symptom burden were strongly associated with allocation to total knee arthroplasty.

The perfect discrimination observed for WOMAC (AUC = 1.000) likely reflects its close relationship with clinical decision-making for surgical allocation, rather than independent predictive performance. As such, WOMAC should be interpreted primarily as a descriptor of group separation rather than as an autonomous prognostic marker.

IL-6 and COMP exhibited moderate-to-good discriminative capability when evaluated separately. These biomarkers may indicate inflammatory activity and cartilage turnover, respectively, and could offer further biological insights beyond age and symptom intensity; nevertheless, their incremental predictive value necessitates validation in multivariable models.

The findings concerning serum vitamin D3 levels corroborate previous research and are a significant determinant in the advancement to total knee arthroplasty for knee osteoarthritis. Reduced vitamin D levels and minor changes in mineral metabolism were correlated with arthroplasty status; however, their independent discriminative efficacy was constrained and warrants careful interpretation.

Calcium, for which a definitive cutoff value remains undetermined, and serum phosphorus, which exhibited normal levels in both cohorts but elevated levels in the TKA group, have demonstrated predictive significance for the necessity of knee arthroplasty when contrasted with the control group. Extensive, prospective, multicenter investigations of substantial scale are required to corroborate the findings.

Prospective longitudinal studies are required to determine whether baseline biomarker profiles can predict future conversion to total knee arthroplasty.

Surgical decision-making in knee osteoarthritis is predominantly influenced by pain intensity, functional impairment, and radiographic joint degeneration. The potential significance of serum biomarkers is not to supplant clinical judgment, but to enhance early risk stratification in diagnostically or therapeutically uncertain situations.

## Figures and Tables

**Table 1:** Summarized biomarker results.

	OR	AUC	*p* Value (AUC)	Sensitivity	Specificity	Cutoff
IL-6	-	0.819	≤0.001	66%	85%	≥4.585 pg/mL
COMP	8.5	0.838	≤0.001	84%	75%	≥11.52 ng/mL
Vitamin D3	0.904	0.683	=0.018	-	-	-
Calcium	-	0.798	≤0.001	-	-	-
Phosphorus	17.569	0.738	=0.002	-	-	-

## Data Availability

The original contributions presented in this study are included in the article. Further inquiries can be directed to the corresponding author.

## Abbreviations

The following abbreviations are used in this manuscript:

KOA	Knee Osteoarthritis
TKA	Total Knee Arthroplasty
WOMAC	Western Ontario and McMaster Universities Osteoarthritis Index
MRI	Magnetic Resonance Imaging
3T MRI	3 Tesla Magnetic Resonance Imaging
IL-6	Interleukin 6
IL-1β	Interleukin 1 beta
TNF-α	Tumor Necrosis Factor alpha
COMP	Cartilage Oligomeric Matrix Protein
CTX-II	C-terminal Cross-linked Telopeptides of Type II Collagen
MMP	Matrix Metalloproteinases
CRP	C-reactive Protein
PTH	Parathyroid Hormone
VDR	Vitamin D Receptor
NSAIDs	Non-Steroidal Anti-Inflammatory Drugs
ROC	Receiver Operating Characteristic
AUC	Area Under the Curve
OR	Odds Ratio
CI	Confidence Interval
SD	Standard Deviation
ANOVA	Analysis of Variance
SPSS	Statistical Package for the Social Sciences
IBM	International Business Machines
MSO	Microsoft Office
χ^2^ (Chi-square)	Chi-square Test
φ (Phi)	Phi Correlation Coefficient
r	Pearson Correlation Coefficient
rs	Spearman Rank Correlation Coefficient
J	Youden Index
